# Case Report: Co-occurring *de novo SHANK3* and *SRCAP* variants in a patient with autoimmune encephalitis and exhibiting Phelan-McDermid syndrome features

**DOI:** 10.3389/fgene.2025.1699311

**Published:** 2025-11-06

**Authors:** Li Li, Jie Zhang, Xiaoyan Shi, Yaqing Huang, Xingzhi Chang, Liya Zhang

**Affiliations:** 1 Department of Neurology, Children’s Hospital of Soochow University, Suzhou, China; 2 Department of Neurology, Peking University First Hospital, Beijing, China

**Keywords:** autoimmune encephalitis, Phelan-McDermid syndrome, SRCAP, SHANK3, variant

## Abstract

Phelan-McDermid syndrome (PMS) is a rare neurodevelopmental disorder caused by a deletion or variant of *SHANK3*. Patients with PMS typically present with global developmental delay, delayed or absent speech, intellectual disability, hypotonia, autism spectrum disorder, behavioral abnormalities, and minor specific dysmorphic features. The *SRCAP* variation is rare and may be associated with chromatin remodeling and neural development. The *SRCAP* and *SHANK3* phenotypes display certain overlapping features, including impaired intellectual and delayed speech development as well as behavioral and psychiatric problems. We report the case of a young male with significant recurrent neuropsychiatric symptoms, developmental regression, and cerebrospinal fluid white blood cell 72/mm^3^. The diagnosis was consistent with antibody-negative autoimmune encephalitis; the patient improved after immunomodulatory treatment. Whole-exome sequencing identified two *de novo* pathogenic frameshift variants, one in *SHANK3* and the other in *SRCAP*, with *SRCAP* being a chimeric variant. Both variants were novel and pathogenic according to the pathogenicity rating provided by the American College of Medical Genetics and Genomics.

## 1 Introduction

*SHANK3* is the main causative gene of Phelan-McDermid syndrome (PMS), and its variants are highly associated with neurodevelopmental, intellectual, psychotic, and mood disorders (Rots et al., 2021). Individuals with PMS have an increased risk of experiencing bipolar disorder, with an associated risk of cognitive and behavioral regression (Breckpot et al., 2016; Denayer et al., 2012; Egger et al., 2016; Serret et al., 2015). *SHANK3* encodes a post-synaptic scaffold protein that plays a key role in synaptic function and neural development (Verhoeven et al., 2013; Costales and Kolevzon, 2016).

*SRCAP* serves as the catalytic core of its eponymous chromatin-remodeling complex, controlling the transcriptional activity of target genes via H2A (Wang et al., 2014). Its expression is detected at low-to-moderate levels across most human tissues (Nagase, 1997). However, heterozygous variants in *SRCAP* are associated with developmental delays, hypotonia, musculoskeletal defects, and behavioral abnormalities (OMIM: 619595). Such variations also cause the Floating-Harbor syndrome (OMIM: 136140), uncommon monogenic conditions with proportionate growth retardation, delayed speech development, and typical facial features. Thus, the phenotypes associated with *SRCAP* and *SHANK3* share some overlapping features.

Autoimmune encephalitis (AE) is an inflammatory disease in which the immune system mistakenly attacks neural structures, leading to cognitive, behavioral, and neurological abnormalities. Antibody-negative AE refers to patients who meet the clinical criteria for AE but in whom well-characterized autoantibodies in serum and cerebrospinal fluid (CSF) are absent, and reasonable alternative causes have been excluded (Dalmau and Graus, 2023). Its etiology is complex and may be related to infection, tumors, or an autoimmune response. Recently, a potential link between neurodevelopmental disorders and immune system abnormalities was identified; however, the specific mechanism underlying this association remains unclear.

This report describes the first case of a patient harboring both *SHANK3* and *SRCAP* variants, co-occurring with autoimmune encephalopathy, and exhibiting features consistent with PMS, thereby expanding the spectrum of PMS-associated *SHANK3* variants. Clinically, when patients with developmental delays and neuropsychiatric symptoms (especially those with known genetic variants) present with acute/subacute symptom exacerbations, comprehensive evaluations including those for AE should be performed as early immunotherapy may improve outcomes.

## 2 Case description

A 15-year-old boy with mild facial deformities: thick eyebrows, long eyelashes, eyelid edema, bulbar nose, vermilion everted lower lip, and triangular face. The knees were excessively extended and valgus, and the walk pattern exhibited a wide-base gait. After experiencing a fever lasting 12 h the patien developed mutism, anxiety, panic, irritability, periodic staring spells, and insomnia. His behavior changed, exhibiting both panic and apathy. The patient was diagnosed with a mental stress disorder, and antipsychotics, benzodiazepines, antidepressants, and anticonvulsants were subsequently administered orally. The patient’s sleep duration increased after 2 days, and he developed somnolence after 1 week. The patient returned to his baseline neuropsychiatric status after several days. The symptoms recurred 1 month later. The patient showed intermittent verbal outbursts, including screaming, and had almost no sleep. In addition, the patient lost the ability to communicate verbally and take care of himself, avoided social contact, lost the ability to chew or swallow, developed intermittent urinary incontinence, and experienced episodes of acute urinary retention. The patient developed spastic dystonia in addition to these degenerative symptoms and was subsequently admitted to the hospital for further diagnosis.

White blood cells, protein, and next-generation sequencing for pathogen detection from CSF studies were normal. Well-characterized autoantibodies were absent in the serum and CSF. Brain magnetic resonance imaging (MRI) revealed no abnormalities. Electroencephalography (EEG) showed slight increases in the diffuse low-medium amplitude slow waves (σ and θ), whereas a moderate level of fast wave rhythm was observed in the bilateral frontal pole, frontal, and frontal midline. Intravenous immunoglobulin (IVIG, 2 g/kg) treatment significantly improved verbal communication, social engagement, sleep, and concentration and decreased agitation. These effects were consistently enhanced over time. However, the patient remained somnolent and unwilling to speak and communicate with others.

After 2 months, the patient was re-admitted to our hospital with a history of fever, vomiting, and diarrhea 1 day before admission. Ten days later, the symptoms recurred, just like he did a month ago. Lumbar puncture, MRI, and video EEG were performed to exclude brain diseases that could be causing these symptoms. The MRI and EEG results were normal. CSF analysis revealed normal lactate and total protein levels, along with a white blood cell count of 72/mm^3^. Autoimmune antibodies in the blood and CSF were negative. Other laboratory tests excluded a rheumatic and neoplastic origin for these findings.

**FIGURE 1:**
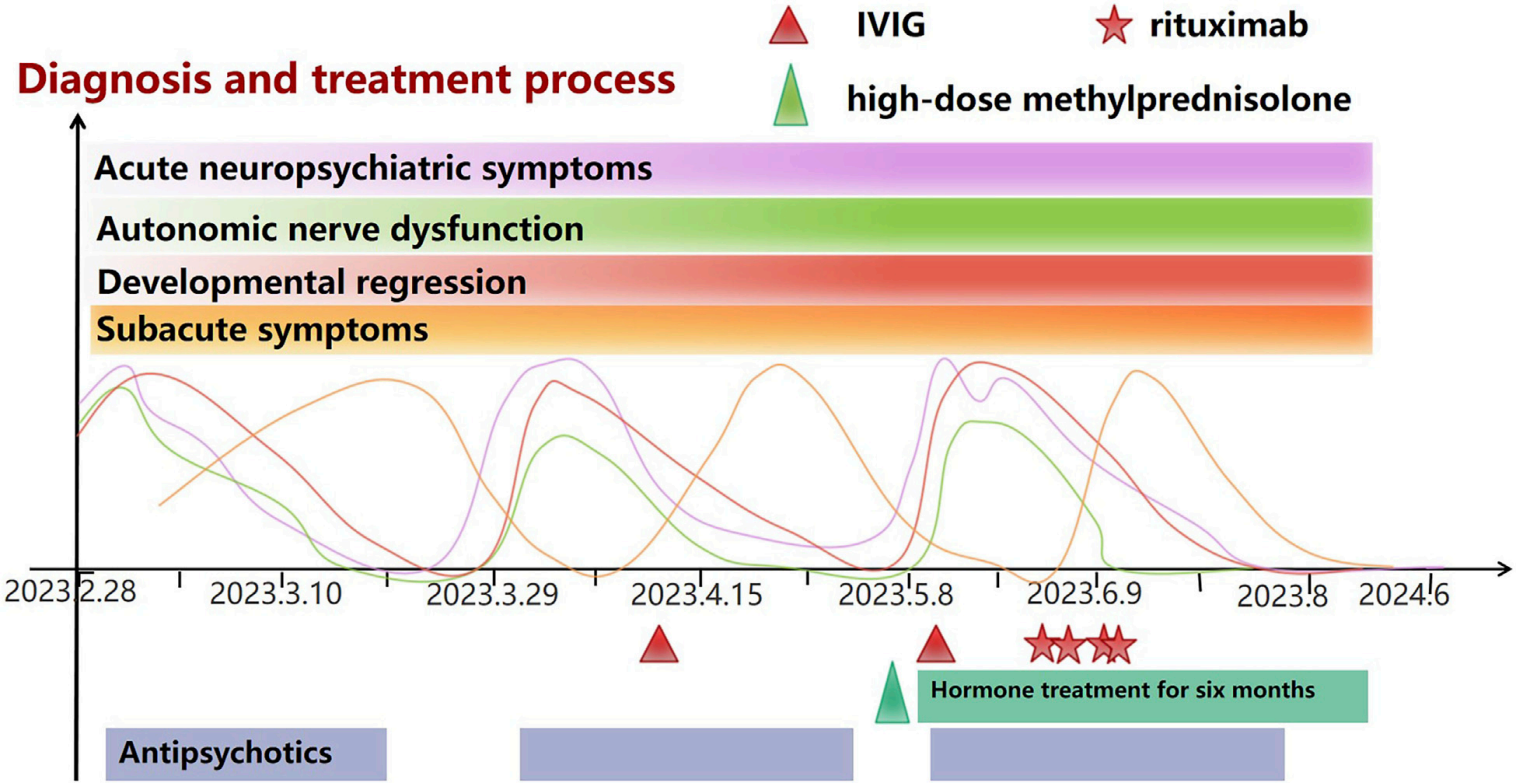
Baseline symptoms: The patient has experienced Global developmental delay from birth to childhood, mild intellectual disability, less sleep, Gait abnormalities, poor coordination, and Hypotonia. From then until nearly 10 years before AE, the patient was in a plateau period and could lead a normal life and study. Acute neuropsychiatric symptoms: Mutism or screaming, anxiety, panic, irritability, periodic staring spells, Insomnia/almost no sleep. Autonomic nerve dysfunction: Intermittent urinary and fecal incontinence, urinary retention, Excessive sweating. Developmental regression: Unable to communicate and unable to take care of himself. Subacute symptoms: Avoided social contact, Preferred confined spaces, Reluctant to speak, indifferent expression, impatient, and short-tempered, Excessive sleepiness.

With antibody-negative AE as a potential diagnosis, the patient was treated with high-dose methylprednisolone therapy (1 g/kg for 5 days, 500 mg/kg for 3 days, followed by full oral dose), which was well tolerated. The symptoms improved but did not resolve completely, and IVIG (1 g/kg) was administered concomitantly, followed by rituximab 2 weeks after hormone therapy. The patient received 2 g/kg IVIG and four weekly doses of rituximab (375 mg/m^2^). Subsequently, an adequate dose of oral prednisone was given and tapered by 2.5 mg per week, with a discontinuation after 6 months. The patient returned to his neuropsychiatric baseline after 1–2 months, at which point all CSF results were normal. Although the patient’s reaction was slower than before the appearance of symptoms, after discharge, he exhibited autistic behavior, was unwilling to socialize, and preferred confined spaces. At follow-up after a year, there was no recurrence of symptoms, return to baseline level, and resolution of autistic behavior (Figure 1).

There was no history of maternal pregnancy complications, and his normal sleep duration had been less than 10 h per day since the neonatal period. At the age of 2 years, the patient walked and drank unsteadily, drooled, and could not speak. After 8 months of rehabilitation treatment, he gained the ability to speak; however, his speech was fast and unclear, and he fell easily while running. At the age of 8–9 years, the patient could walk steadily and run, but he was unable to run fast or jump on both feet, and he had poor coordination. The Wechsler Intelligence test for children yielded intelligence quotients of 65 and 75 at the ages of 5 and 7 years, respectively. At the time of this report, the patient was attending a vocational junior high school with low grades in mathematics and physics.

Whole-exome sequencing was performed to investigate the etiology of complex AE and early-life developmental delay. DNA was extracted from parents and proband samples, using QIAamp® Blood Mini Kit (Qiagen, Hilden, Germany) following the manufacturer’s protocol. The trioWES libraries were quantified by qPCR and sequenced using Illumina NovaSeq6000 (Illumina, San Diego, CA, United States) with 150 base-paired end reads. Coverage for these patients was at least 98.97% at a 20× depth threshold. The sequencing reads were aligned to the human reference genome (hg38/GRCh38) using Burrows-Wheeler Aligner tool (Li and Durbin, 2009) and PCR duplicates were removed by using Picard (http://picard.sourceforge.net/). Verita Trekker® Variants Detection System by Berry Genomics and the third-party software GATK (Do Valle et al., 2016) (https://software.broadinstitute.org/gatk/) were employed for variant calling. Variant annotation and interpretation were conducted by ANNOVAR[4] and the Enliven® Variants Annotation Interpretation System authorized by Berry Genomics. Annotation databases mainly included human population databases (gnomAD, ExAC, 1,000 Genome Project, ESP), silico prediction algorithms (SIFT, FATHMM, REVEL, CADD, SPIDEX) and disease and phenotype databases (OMIM, ClinVar, HGMD). The variants were classified according to the American College of Medical Genetics and Genomics (ACMG) guidelines for interpretation of genetic variants (Richards et al., 2015).

**FIGURE 2:**
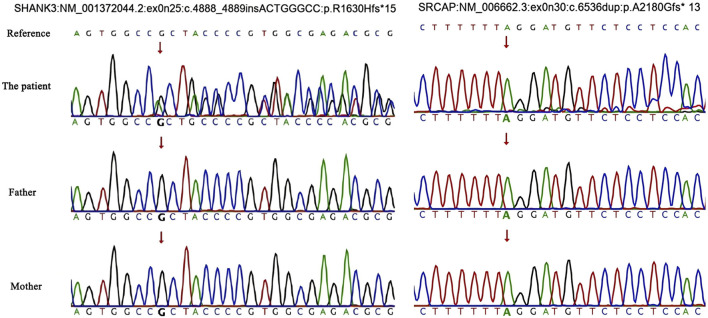
Single-nucleotide variations and insertion–deletions. An arrow indicates the target nucleotide.

**FIGURE 3:**
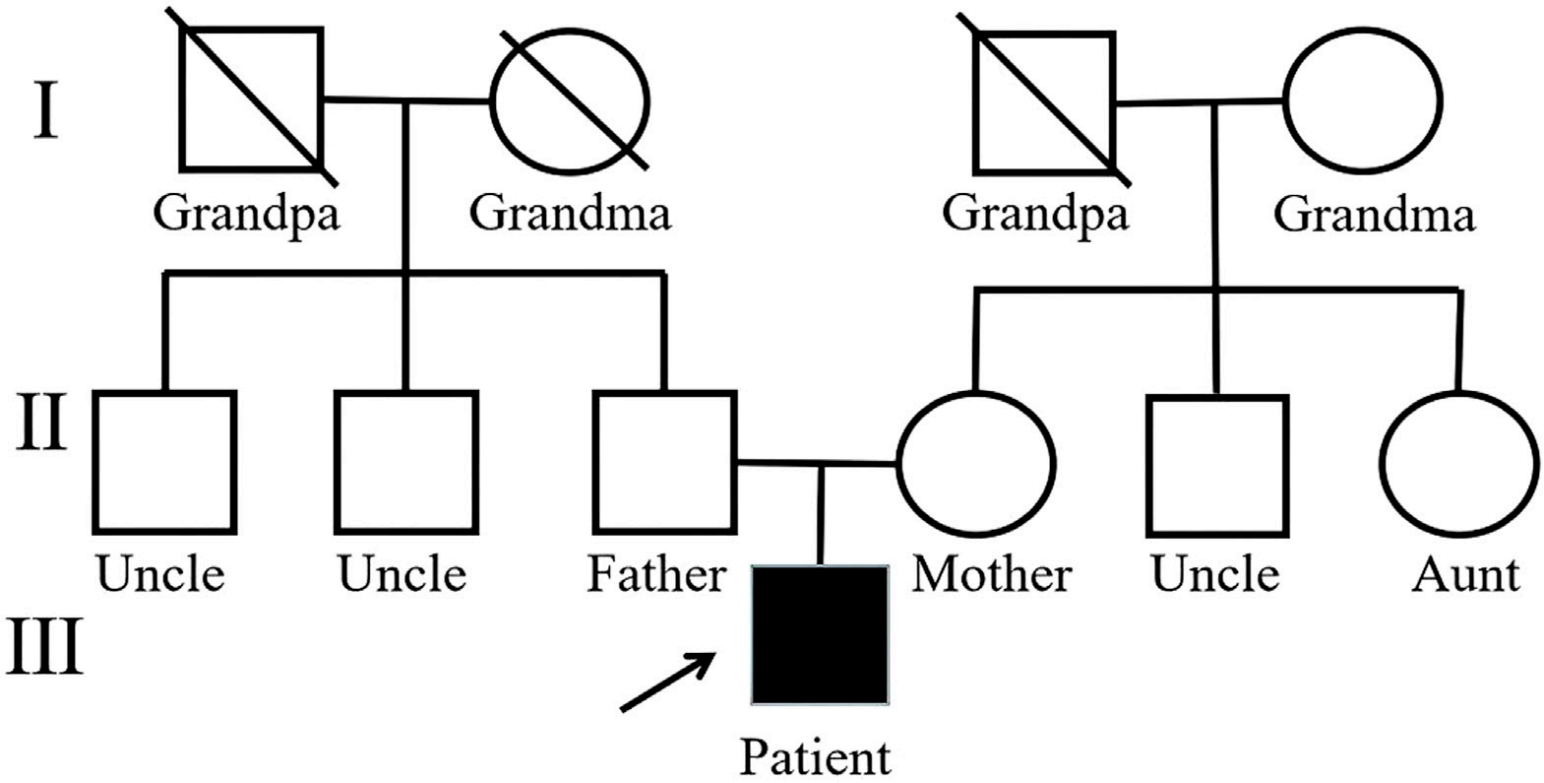
Pedigree of the proband.

Two *de novo* pathogenic frameshift variants were identified, one in exon 25 of *SHANK3* and another in exon 30 of *SRCAP.* The variation rate of *SRCAP* is 15%, which is suspected to be a chimeric variant. Neither of these variants has been previously reported. Single-nucleotide variant and insertion–deletion results showed that the patient was heterozygous for *SHANK3* c.4888_4889insACTGGGCC (p.R1630Hfs*15) and chimeric variant for *SRCAP* c.6536dup (p.A2180Gfs*13), whereas no variants were identified in either of his parents (Figures 2, 3). Neither variants have been recorded in the gnomAD, 1,000 genome Project, or Human Exon database (ExAC) (PM2_Supporting). They were classified as Pathogenic according to the American College of Medical Genetics and Genomics (ACMG) guidelines (Table 1).

**TABLE 1:** Classification of identified variants according to ACMG/AMP guidelines.

Gene	*SHANK3*	*SRCAP*
HGVS Nomenclature	NM_001372044.2: c.4888_4889ins ACTGGGCC:p.R1630Hfs*15	NM_006662.3: c.6536dup p.A2180Gfs*13
Genomic Coordinate (GRCh38)	chr22:50730814-50730814	chr16:30733929-30733929
ACMG Classification	Pathogenic	Pathogenic
ACMG Evidence Codes	PVS1_Strong Frameshift variant located in last exon of gene and predicted to not undergo NMD, variant removes >10% of protein	PVS1 Frameshift variant predicted to undergo NMD and exon is present in biologically-relevant transcript
	PS2: *de novo* with confirmed parental relationships and phenotype highly specific for gene	PS2: *de novo* with confirmed parental relationships and phenotype highly specific for gene
	PM2_Supporting Absent in gnomAD, 1,000 genome Project and Human Exon database (ExAC)	PM2_Supporting Absent in gnomAD, 1,000 genome Project and Human Exon database (ExAC)
	PS2+PVS1_Strong+PM2	PVS1+PS2+PM2

**FIGURE 4:**
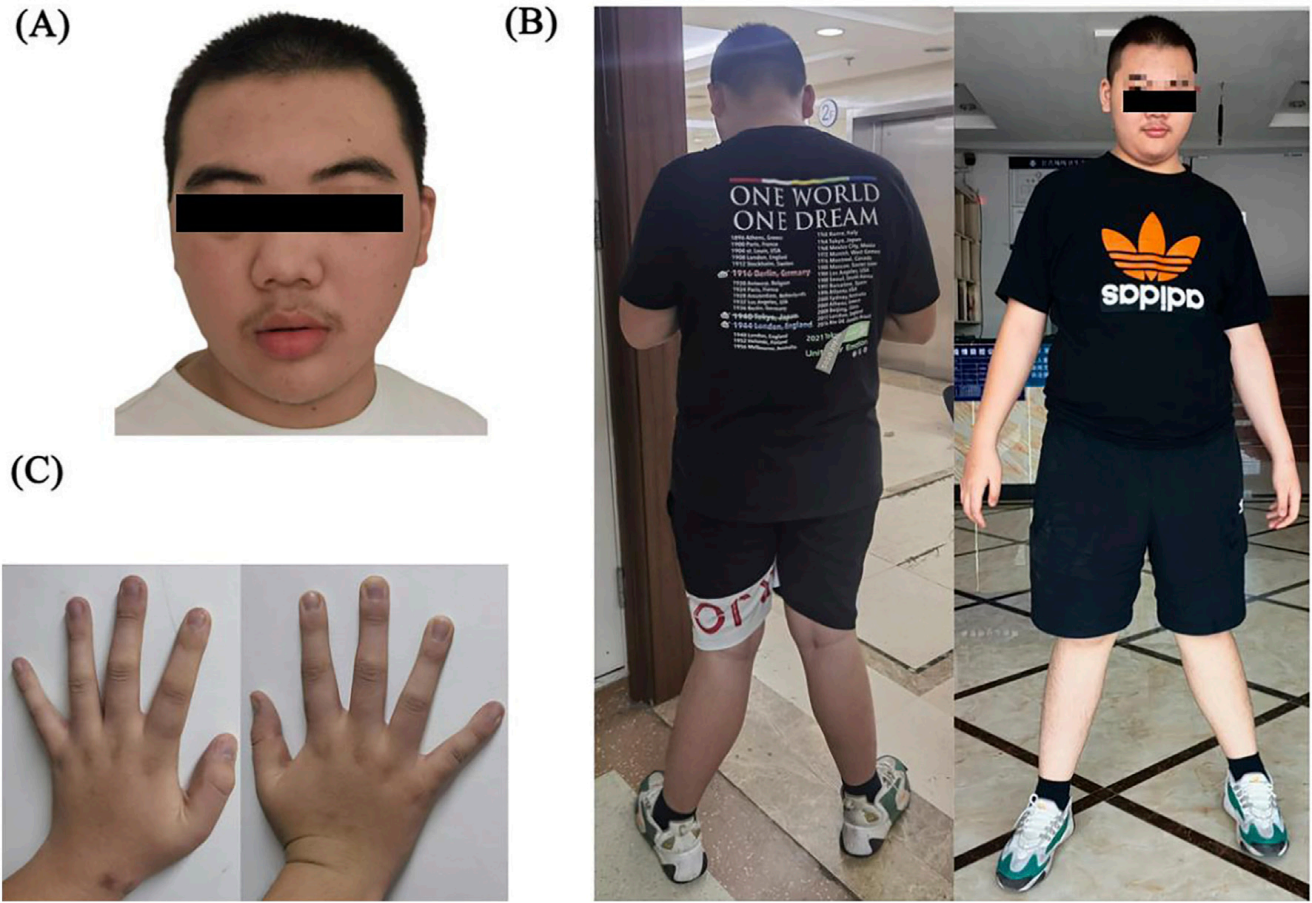
**(A)** Facial dysmorphism: thick eyebrows, prominent supraorbital crest, ptosis, long eyelashes, eyelid edema, hypertelorism, narrow palpebral fissure, broad and depressed nasal bridge, bulbar nose, flattened zygoma, everted vermilion lower lip, and triangular face. **(B)** Both knees were excessively extended and valgus, and the walk pattern exhibited a wide-base gait. **(C)** Broad fingertips, short fingers and metacarpal bones, and hypoplastic finger- and toenails.

Facial dysmorphism, consistent with *SHANK3* and *SRCAP* variations, is characterized by thick eyebrows, prominent supraorbital crest, ptosis, long eyelashes, eyelid edema, hypertelorism, narrow palpebral fissure, broad and depressed nasal bridge, bulbar nose, flattened zygoma, everted lower lip vermilion, and triangular face. When standing, both knees are excessively extended and valgus, accompanied by a walk pattern characterized by a wide-base gait. The fingertips are broad, the fingers and metacarpal bones are short, and the finger- and toenails are hypoplastic (Figures 4A–C). Otorhinolaryngologic examinations revealed a low-hanging columella.

This WES test also identified six other related variants: *SPG11, INTS1, AUTS2, PEX1, HERC2, and XYLT1*, which are partially related to the clinical phenotypes of the subjects. However, there is insufficient evidence of genetic pathogenicity, and further clinical attention is needed.

## 3 Discussion

According to the 2023 criteria for probable antibody-negative autoimmune pediatric encephalitis (Dalmau and Graus, 2023) diagnosis requires: 1) subacute onset (<3 months) of memory deficits, altered mental status, or psychiatric symptoms. 2) at least one of new focal CNS deficits, unexplained seizures, CSF pleocytosis, or MRI findings suggestive of encephalitis. 3) absence of well characterized autoantibodies in serum and CSF. 4) reasonable exclusion of well-defined syndromes of AE and alternative causes. This case report describes the case of a pubertal male with a history of mild developmental delay who developed a neuropsychiatric syndrome with regression after an acute infection. The patient presented with subacute and recurrent behavioral symptoms, with a white blood cell count of 72/mm^3^ in CSF and EEG showed slight increases in slow waves. Symptoms improved after immunomodulatory therapy. Overall, this case fulfilled the dissemination criteria for antibody-negative AE. Whole-exome sequencing revealed pathogenic frameshift variants in *SHANK3* and *SRCAP,* the variation rate of *SRCAP* is 15%, which is suspected to be a chimeric variant. Consistent with these variants, the patient presented with speech delay, cognitive and behavioral regression, behavioral abnormalities, and regression.

*SHANK3* variants are associated with an increased risk of psychiatric abnormalities and regression, raising the question of whether the deterioration in the present case occurred as a consequence of an acute incident (such as infection or antibody-negative AE or whether it was inherent to PMS. We believe that both factors were considered. Emerging evidence indicates that individuals with PMS may experience severe neuropsychiatric symptoms and loss of skills occurring in adolescence and adulthood (Kolevzon et al., 2019; Kohlenberg et al., 2020). Clinical presentations included features of bipolar disorder, catatonia, psychosis, and loss of skills. Triggers may include infections, changes in hormonal status, and stressful life events. This stage is highly sensitive to recurrent infections (Dille et al., 2023). But reports of patients with PMS have shown no evidence of pathological findings in routine CSF testing (Bey et al., 2020; Jesse et al., 2023). In this case, CSF pleocytosis fulfilled the diagnostic criteria for antibody-negative AE. In addition, inflammatory stimuli and sex hormones can regulate *SHANK3* expression (Zhao and Lukiw, 2018; Berkel et al., 2018).

Another question raised by this case is whether the occurrence of *SHANK3* variants and AE was a coincidence or whether both conditions share a common underlying pathophysiological mechanism. Currently, no conclusive evidence supports a direct causal relationship or a common underlying mechanism. However, from a neuroscience and immunological perspective, some potential indirect associations can be speculated. *SHANK3* has been extensively studied for its role in post-synaptic function (Sala et al., 2015). It protein product acts as a structural scaffold within the post-synaptic density, comprising six distinct domains that mediate connections between the actin cytoskeleton and multiple membrane-bound and cytoplasmic proteins, such as AMPA, NMDA, and mGluR receptors, as well as PSD-95. Through these protein-protein interactions, *SHANK3* promotes synaptogenesis and plasticity, regulates dendritic spine morphology, and facilitates the transport, anchoring, and proper aggregation of glutamate receptors and glutamatergic synaptic adhesion molecules (Verpelli et al., 2012). Autoantibodies against the synaptic proteins supported by its scaffolding have also been implicated in the onset of AE (Vova and Howarth, 2023). In addition to its post-synaptic roles, *SHANK3* is also expressed pre-synaptically during development, regulating presynaptic NMDA receptor levels at axon terminals (Halbedl et al., 2016). So, we speculate that when *SHANK3* becomes inactive, it first directly disrupts postsynaptic NMDA, AMPA and mGluR receptors, leading to abnormal exposure and increasing their immunogenicity, In addition, the presynaptic regulatory effect of *SHANK3* on neurotransmitter release is also simultaneously impaired. Therefore, patients with *SHANK3* variations are more prone develop to AE. Patients diagnosed with neurodevelopmental conditions (including autism spectrum disorder) frequently demonstrate immune dysregulation, which may establish a predisposing environment for AE (Estes and McAllister, 2015). Further functional research is needed to explore this connection.

The proteins encoded by *SRCAP* are primarily involved in chromatin remodeling and gene expression regulation, with their core function being to influence gene transcription by modulating chromatin structure. Currently, no direct evidence supports that *SRCA* variants are associated with the pathological mechanism of AE, however, the role of *SRCAP* in chromatin remodeling and gene expression regulation may indirectly affect the function of the immune system. For example, Chen et al. (2017) discussed the role of chromatin remodeling in the immune system, including T-cell differentiation, inflammatory responses, and potential mechanisms in autoimmune diseases. Wang et al. (2016) explored the role of chromatin remodeling in autoimmune diseases, including systemic lupus erythematosus, systemic sclerosis, and multiple sclerosis. Liu et al. (2024) demonstrated that chromatin remodeling inhibits inflammation in animal models of experimental autoimmune encephalomyelitis and colitis. At present, we are still unable to confirm whether there is a relationship between *SRCAP* and AE. In the future, we will carry out functional research to verify.

Truncating variants in the last two exons (exons 33 and 34) of the *SRCAP* cause the neurodevelopmental disorder Floating-Harbor syndrome (FLHS). Proximal truncation variations of the FLHS locus can lead to non-FLHS *SRCAP*-related NDD, accompanied by behavioral and mental problems, non-specific deformity features, musculoskeletal problems and hypotonia. In this case, the *SRCAP* variation of the patient is located in exon 30, which can cause non-FLHS *SRCAP*-related symptoms. The variation ratio is low (15%), and normal heterozygous variations are generally around 50%. It is considered not a heterozygous variation and is suspected to be chimerism. Chimeric variation (commonly known as somatic chimeric phenomenon) refers to a postzygotic genetic variation that exists only in some cells of the patient, rather than a germline variation that exists in all cells. The clinical manifestations of chimeric variations vary due to different variation ratios. Generally, the higher the chimeric ratio, the more obvious the abnormal phenotype. Therefore, in terms of phenotypes, the PMS-related phenotypes caused by non-FLHS *SRCAP* and *SHANK3* have some overlapping characteristics, and both phenotypes are present in this patient.

There are currently no relevant literature reports on whether there is a synergy effect between *SHANK3* and *SRCAP*. *SHANK3* is a core scaffold protein that is crucial for the structural and functional integrity of excitatory synapses. *SRCAP*, as a chromatin remodeling factor, which places upstream of *SHANK3* in the regulatory hierarchy. We speculate that the defect of *SRCAP* function may lead to a decrease in *SHANK3* expression, generating a synergistic effect. Future We propose to introduce both the *SHANK3* and *SRCAP* variants (alone and in combination) into a neuronal cell model and assess the subsequent transcriptomic and proteomic profiles, specifically focusing on pathways related to neuroinflammation and synaptic function. This would help determine if the combined variations lead to a unique or a synergistic effect.

According to a recent publication (Bey et al., 2020), four teenage girls diagnosed with PMS exhibited subacute neuropsychiatric deterioration accompanied by regression in the behavioral and developmental domains. Unlike the present case, diagnostic studies were generally uninformative. However, each of the treating physicians independently considered AE as a potential etiology for the regressive episodes exhibited by the patients. Further, immunomodulatory therapies resulted in symptom improvement in all cases. Current PMS management guidelines (Srivastava et al., 2023) recommend trial immunomodulation in patients with abrupt neurological changes (including seizures or regression) and when comprehensive workup remains unremarkable, indicating potential seronegative AE. However, further research is needed to characterize the neuropsychiatric aspects of PMS that are immunologically mediated.

Our article has limitations. Due to limited conditions, high-resolution CMA, cytogenetics and MLPA techniques were not performed for the first time to eliminate microdeletions and identify other rearrangements. Second, laboratory functional studies or in-depth mechanism experiments were not conducted to verify the pathogenicity or effect of the *SHANK3* and *SRCAP* variants. In this case, we found that both the *SHANK3* gene and *SRCAP* were *de novo* truncated mutations. There have been many reports of diseases caused by *SHANK3* (Arons et al., 2012; Durand et al., 2012; Cochoy et al., 2015) and *SRCAP* (Rots et al., 2021; Franz et al., 2020) truncation mutations in the past, and these have been confirmed by laboratory functional studies. Given that this specific *de novo* truncation variant is highly penetrative and has been confirmed as a pathogenic mutation, we are highly convinced that it is the primary genetic cause of the patient’s illness. Furthermore, we reviewed the WES no evidence of a large deletion. In the future, we plan to refine CMA or karyotypes to rule out microdeletions or gene rearrangements and apply them in similar cases. Conduct laboratory functional studies or in-depth mechanism experiments to verify the pathogenicity or impact of SHANK3 and SRCAP variations.

## 4 Conclusion

In conclusions, this case is the first to report dual pathogenic variants *SHANK3* and *SRCAP* co-occurring with autoimmune encephalopathy, expanding the spectrum of *SHANK3* variants associated with PMS. This case underlines the importance of comprehensive diagnostic evaluation in patients with developmental delays. When neuropsychiatric symptoms occur, assessments should include standard neuropsychological tests, cerebrospinal MRI, electroencephalography, lumbar puncture, and in selected cases, genetic analysis.

## Data Availability

All relevant data is contained within the article. The original contributions presented in the study are included in the article, further inquiries can be directed to the corresponding authors.
